# Long-term in vitro culture of *Plasmodium vivax* isolates from Madagascar maintained in *Saimiri boliviensis* blood

**DOI:** 10.1186/s12936-017-2090-7

**Published:** 2017-11-03

**Authors:** Rajeev K. Mehlotra, D’Arbra Blankenship, Rosalind E. Howes, Tovonahary A. Rakotomanga, Brune Ramiranirina, Stephanie Ramboarina, Thierry Franchard, Marlin H. Linger, Melinda Zikursh-Blood, Arsène C. Ratsimbasoa, Peter A. Zimmerman, Brian T. Grimberg

**Affiliations:** 1 Center for Global Health and Diseases, Case Western Reserve University School of Medicine, Cleveland, OH 44106-4983 USA; 2 Oxford Big Data Institute, Nuffield Department of Medicine, University of Oxford, Oxford, UK; 3 National Malaria Control Programme, Ministry of Health, Antananarivo, Madagascar; 4 Faculty of Sciences, University of Antananarivo, Antananarivo, Madagascar

**Keywords:** Madagascar, In vitro culture, *Plasmodium vivax*

## Abstract

**Background:**

*Plasmodium vivax* is the most prevalent human malaria parasite and is likely to increase proportionally as malaria control efforts more rapidly impact the prevalence of *Plasmodium falciparum*. Despite the prominence of *P. vivax* as a major human pathogen, vivax malaria qualifies as a neglected and under-studied tropical disease. Significant challenges bringing *P. vivax* into the laboratory, particularly the capacity for long-term propagation of well-characterized strains, have limited the study of this parasite’s red blood cell (RBC) invasion mechanism, blood-stage development, gene expression, and genetic manipulation.

**Methods and results:**

Patient isolates of *P. vivax* have been collected and cryopreserved in the rural community of Ampasimpotsy, located in the Tsiroanomandidy Health District of Madagascar. Periodic, monthly overland transport of these cryopreserved isolates to the country’s National Malaria Control Programme laboratory in Antananarivo preceded onward sample transfer to laboratories at Case Western Reserve University, USA. There, the *P. vivax* isolates have been cultured through propagation in the RBCs of *Saimiri boliviensis*. For the four patient isolates studied to-date, the median time interval between sample collection and in vitro culture has been 454 days (range 166–961 days). The median time in culture, continually documented by light microscopy, has been 159 days; isolate AMP2014.01 was continuously propagated for 233 days. Further studies show that the *P. vivax* parasites propagated in Saimiri RBCs retain their ability to invade human RBCs, and can be cryopreserved, thawed and successfully returned to productive in vitro culture.

**Conclusions/significance:**

Long-term culture of *P. vivax* is possible in the RBCs of *Saimiri boliviensis*. These studies provide an alternative to propagation of *P. vivax* in live animals that are becoming more restricted. In vitro culture of *P. vivax* in Saimiri RBCs provides an opening to stabilize patient isolates, which would serve as precious resources to apply new strategies for investigating the molecular and cellular biology of this important malaria parasite.

**Electronic supplementary material:**

The online version of this article (10.1186/s12936-017-2090-7) contains supplementary material, which is available to authorized users.

## Background

*Plasmodium vivax* threatens the public health of over 2.5 billion people in 95 countries around the world [1, 2]. While the knowledge about *P. vivax* has been significantly advanced through in vivo infections of humans [3–7] and non-human primates [8–10], incentives to develop in vitro culture systems arise from ethical concerns and the costs linked to propagation of malaria parasites in these hosts [11–13]. In contrast to *Plasmodium falciparum* [14] and other human/simian [15–19] malaria parasite species, laboratory methods for culturing *P. vivax* have lagged behind, and have limited studies on the molecular and cellular parasitology of this parasite. Therefore, significant research interest has been focused on improving in vitro culture of *P. vivax* [20–23].

The goal of in vitro culture studies is easily understood from the success that has been demonstrated in the laboratory with *P. falciparum* over the past 40 years [14]. Through the availability of a robust approach for culturing *P. falciparum*, it has been possible to adapt parasite strains for stable in vitro culture from infected human blood samples and from isolates propagated in non-human primates since the mid-1970s [24–26]. Parasites have been grown with sufficient viability in the laboratory to perform genetic crosses and to isolate progeny to enable genetic linkage studies that have identified genes linked to specific parasite phenotypes [27–30]. It has been possible to identify components of the parasite’s red blood cell (RBC) invasion mechanism [31–33], and to examine mechanisms of resistance against a wide range of anti-malarial drugs [34–36]. Rounding out the achievements arising from reliable culture of *P. falciparum* is the ability to perform genetic manipulation of the parasite to verify genetic underpinnings of the parasite’s complex biology [22, 37–39]. Much of this has required development of methods for long-term culture, storage, and rejuvenation of well-characterized parasite strains.

Various methods have been described and reviewed since Bass and Johns first attempted to culture *P. vivax* in 1912 [40]. These approaches have employed a number of strategies for preparing parasites for in vitro culture, adjusting the culture media and varying concentrations of human serum ([41–50]; reviewed in [20, 21]). Acknowledging the *P. vivax* host reticulocyte cell preference, considerable attention has focused on reticulocyte enrichment methods and supplementation of cultures with those cells [41, 50–56]. Despite progress, observations published and unpublished have acknowledged the challenges still experienced in attempts to culture this parasite. Additionally, attempts to develop a durable system for long-term in vitro culture of *P. vivax* remain tentative.

Many of these challenges can be overcome by use of non-human primate models, such as the Bolivian squirrel monkey, *Saimiri boliviensis*, which allows optimized blood-stage *P. vivax* infections with blood draws timed to enrich individual life cycle stages, allowing increased association of identified proteins with individual developmental stages and disease processes [57, 58]. Additionally, studies using *Plasmodium cynomolgi* propagated in Southeast Asian macaques have provided opportunity to perform valuable heterologous comparative RBC invasion analyses of human RBCs with *P. vivax* [59]. Despite these efforts to propagate these parasites in live animals, there are efforts to eliminate (chimpanzees [60]) or curtail (monkeys [61, 62]) these animal models, and repository supplies of historically available resources are approaching a critically low level.

A goal of the current studies in Madagascar is to collect *P. vivax* isolates from infected people and prepare them for in vitro growth and red cell invasion experiments. These studies are performed in follow-up to the observation that *P. vivax* demonstrates significant capacity to infect Duffy-negative individuals in Madagascar [63], and the observation of new *P. vivax* genes and genetic variation [64, 65]. As a first step toward this goal, a system has been established for collection and cryopreservation of infected blood samples from people living in rural communities significantly removed from laboratory facilities, followed by long-term propagation in an in vitro culture system. Taking the lead from past and present efforts, this culture system uses RBCs from *S. boliviensis*, as it has been used for *P. vivax* short-term in vitro culture [45], and has also been used extensively to support parasite propagation in live animals [57, 66–70]. Although a number of studies have included efforts to enrich reticulocytes, the demonstrated target cells for *P. vivax* [41, 51, 52, 54–56], the in vitro culture system evaluated here did not include reticulocyte enrichment. The methods described here enable in vitro propagation, expansion, and cryopreservation of patient isolates for further studies.

## Methods

### Study site and protocol guidelines

This study was conducted in the western highlands fringe region of Madagascar, in the foothills between the central highlands and the western tropical coastal zone. This area is endemic for both *P. falciparum* and *P. vivax* malaria [63], and shows distinct malaria seasonal trends that peak from April to May annually [71].

Longitudinal surveillance was set up in partnership with local doctors at the three Ampasimpotsy health facilities [GPS locations: − 19.19685, 46.1390 (north); − 19.24525, 46.18026 (center); − 19.37066, 46.11724 (south)]. These facilities are rural clinics serving catchment populations of 1500–3000 in the Tsiroanomandidy health district [72]. All patients reporting to a clinic with fever were screened for malaria using the SD Bioline Malaria Ag P.f/Pan rapid diagnostic test (RDT) and light microscopy by a WHO-certified technician. Any patients with *P. vivax* were invited to participate in the present study. Patients were enrolled following informed consent by a local doctor qualified in NIH Human Subjects Training. A parent or guardian provided written informed consent on behalf of participants under the age of 18 years. This study was conducted following a protocol approved by the University Hospitals of Cleveland Institutional Review Board (#09-13-01), the Division of Microbiology and Infectious Diseases/NIAID/NIH (#13-0067), and the Madagascar Ministry of Health Ethics Committee (#099). All malaria infections were treated by the health practitioner with an age-adjusted course of artesunate-amodiaquine, in accordance with the Madagascar Ministry of Health guidelines [73].

### Sample collection, processing and storage

A 7–10 mL venous blood sample was drawn into a K^+^-EDTA and/or Na^+^-heparin vacutainer and stored at 4 °C up to 24 h prior to cryopreservation. Cryopreservation of *P. vivax*-infected blood samples closely followed the method described by J. Normark [74]. The blood sample was mixed with an equal volume of Glycerolyte 57 solution and aliquoted into 2-mL tubes for cryopreservation. The tubes were stored in a −20 °C freezer for 24 h and subsequently transferred into a liquid nitrogen dry shipper. Cryopreserved samples were shipped from Madagascar to the U.S. over a 10-day period. The samples were unpacked onto dry ice to minimize thawing during transfer to longer-term storage in a liquid nitrogen freezer.

### In vitro cultivation of *Plasmodium vivax* patient isolates

In vitro culture of the *P. vivax* isolates described herein was initiated for four different *P. vivax*-infected patient blood samples. These included patient sample Ext30703 (isolate—AMP2014.01, collected in EDTA), patient sample 1040902 (AMP2014.02, collected in heparin), patient sample Ext2838 (AMP2016.01, collected in EDTA) and patient sample Ext3276 (AMP2016.02, collected in EDTA). These *P. vivax* isolates were cultured in AIM V serum-free medium (Gibco), containing l-glutamine, streptomycin sulfate at 50 µg/mL, and gentamicin sulfate at 10 µg/mL. AIM V medium was developed in 1987 for generating lymphokine-activated killer cells to support adoptive immunotherapy clinical trials [75–77] and to culture/proliferate macrophages [78] and lymphocytes [79, 80]. The medium is substantially equivalent to Dulbecco’s Modified Eagle Medium (DMEM), used broadly for tissue and cell culture processing applications (manufacturer’s notes). In the present study, AIM V medium was supplemented with 2.4 mM l-glutamine, 10 mM HEPES (pH 7.4), and 0.2 mM hypoxanthine. 10-mL aliquots were stored in 15-mL conical base centrifuge tubes at −80 °C. Immediately before using, the medium-containing tube was completely thawed and heat-inactivated, pooled human AB serum (Gemini Bio-products) was added at 10% final concentration. This complete AIM V medium was kept at 37 °C in a 10% CO_2_ incubator until use.

The cryopreserved *P. vivax*-infected patient blood samples were thawed using a modified, decreasing NaCl concentration gradient (12, 1.6, and 0.9%) method [81]. After the final NaCl treatment, the blood sample pellet volume was measured (30–80 μL) and re-suspended in 5 mL of complete AIM V medium in a 6-well tissue culture plate. Two mL whole blood from Bolivian squirrel monkeys (*S. boliviensis*), collected in EDTA, was obtained from the Biologics Production Program, Michale E. Keeling Center for Comparative Medicine and Research, The University of Texas MD Anderson Cancer Center (Protocol Number: 00000451-RN01-AR001). The blood was shipped on ice overnight, and was leukocyte depleted using CF11 (Whatman) cellulose powder-filled plastic syringe columns immediately after it was received. Following depletion, it was stored at 4 °C for 2–3 weeks in complete malaria culture medium (CMCM) [82]; 2 mL CMCM was added to 1 mL RBCs. The leukocyte-depleted Saimiri blood (e.g. 1746 cells counted, no WBCs observed) was added to the complete AIM V medium containing *P. vivax*-infected patient blood pellet to achieve 4% haematocrit. Parasite cultures were maintained at 37 °C in 10% CO_2._ The culture medium was changed daily using freshly prepared complete medium.

Lysis was observed in the culture every 96–120 h. At that time, the whole content of the well was transferred to a 15-mL conical tube and centrifuged (1000 rpm, 5 min). The pellet was measured, re-suspended in fresh complete medium (5 mL) in a new well, and Saimiri RBCs were added to restore 4% haematocrit. Generally, lysis was approximately 50%, as the pellet was measured to be approximately 100 µL, and did not appear to increase as a function of blood storage time at 4 °C.

### Microscopy

Standard blood smear microscopy was performed to monitor parasite growth. Briefly, thin smears were prepared by spreading 2 µL of resuspended cultures on a glass slide and were fixed in 100% methanol. Fixed slides were stained with 4% Giemsa (Sigma-Aldrich), diluted with 1× buffered distilled water pH 7.4, for 30 min, and examined using oil immersion (100×). Parasitaemia was estimated by counting a total of 1000 RBCs from 10 to 20 fields, each with 50–100 RBCs. On a few occasions, a second slide was made and evaluated. The percent of infected RBCs was determined by enumerating the number of infected RBCs in relation to the number of uninfected RBCs ([No. infected RBCs/Total No. RBCs counted] × 100 = Percent Infected RBCs). Reticulocytes in Saimiri blood were enumerated using Retic-Chex^®^ Stain (Streck) following the manufacturer’s instructions.

### Preparation of DNA template

Genomic DNA was extracted using a QIAamp DNA Micro Kit (QIAGEN) according to the manufacturer’s protocol. Extracted samples included *P. falciparum*, *P. vivax*, *Plasmodium malariae*, and *Plasmodium ovale*-infected blood samples that served as polymerase chain reaction (PCR) controls; blood samples were provided by the Malaria Research and Reference Reagent Resource Center (MR4; now merged with BEI Resources) and Dr. W. E. Collins (Centers for Disease Control and Prevention). Additionally, DNA was extracted from 20 to 100 µL of each *P. vivax*-infected Madagascar patient sample and *P. vivax* in vitro culture.

### Molecular diagnosis of *Plasmodium* species infection

PCR-based *Plasmodium* species diagnosis employed a ligase detection reaction-fluorescent microsphere assay (LDR-FMA). All methods for PCR amplification of small sub-unit rRNA target sequences and *Plasmodium* species-specific detection by LDR-FMA have been described in detail by McNamara et al. [83]. Species-specific fluorescence data were collected using the Bio-Plex Manager 3.0 software (Bio-Rad).

### DNA sequence analysis of polymorphic *Plasmodium vivax*-specific genes

To have a peek at potential strain complexities of the *P. vivax* field isolates, nested PCR amplification was performed to amplify *P. vivax* Duffy binding protein (*PvDBP*) and apical membrane antigen-1 (*PvAMA*-*1*) gene sequences. Nested PCR amplification reactions (25 μL) were performed (0.2 μM for each of the appropriate upstream (Up) and downstream (Dn) primers, 10 mM Tris–HCl (pH 8.3), 1.5 mM MgCl_2_, 50 mM KCl, 0.01% gelatin, 50 μM for each dATP, dGTP, dCTP, and dTTP, 2.5 units of thermostable DNA polymerase, and 3 μL of DNA template) for both gene sequences using the primer pairs provided in Additional file 1: Table S1. *PvDBP* PCR conditions for both nest 1 and nest 2 were: 95 °C 1 min; 95 °C 30 s, 58 °C 30 s, 72 °C 45 s (× 39); 72 °C 4 min. *PvAMA*-*1* PCR conditions were according to Moon et al. [84]. After 2% agarose gel electrophoresis (1 × Tris–borate–EDTA buffer) and staining with SYBR Safe DNA Gel Stain (Invitrogen), the *PvDBP* and *PvAMA*-*1* nest 2 reaction products were observed to be of 518 and 395 bp in length, respectively.

Sanger sequencing of *PvDBP* and *PvAMA*-*1* nest 2 amplicons was performed using a modified Applied Biosystems BigDye^®^ Terminator v3.1 Cycle sequencing kit protocol. Sequencing reactions were performed on the ABI 3730 DNA Analyzer system (Applied Biosystems). Sequences were analysed using Sequencher v5.2 (Gene Codes Corporation) and Geneious 7.0.6 software (Biomatters).

### Saimiri-adapted *Plasmodium vivax* invasion of human RBCs

To test whether Saimiri blood-adapted *P. vivax* could successfully invade human RBCs, a second culture of isolate AMP2014.01 was initiated from a duplicate cryopreserved patient blood sample vial 961 days after collection. The culture conditions, including medium, Saimiri RBCs, incubation temperature and atmosphere, of the second culture were the same as described above. The culture in Saimiri RBCs was maintained for 25 days ahead of performing the human RBC invasion experiment. Over these 25 days, the culture medium was changed daily using freshly prepared complete medium, and Saimiri RBCs were added 5 times to maintain 4% haematocrit. The average parasitaemia during this time was 0.325%. The parasitaemia on the day that the invasion experiment was initiated was 0.3%.

For the human RBC invasion experiment, the blood was drawn from a local donor of European descent carrying FY*B/*B genotype. The blood was leukocyte depleted using CF11 (Whatman) cellulose powder-filled plastic syringe columns, before use in the experiment.

In this invasion experiment, Saimiri and human RBCs without parasites were used as controls. Each experimental and control condition was plated in triplicate in a 96-well plate. The total volume per well was 180 μL. For ensuring the reintroduction of Saimiri blood-adapted *P. vivax* to human RBCs, each experimental well had 4% haematocrit (total 7.2 μL RBCs) with an estimated parasite:target ratio of 1:9 (0.72 μL of infected Saimiri blood and 6.48 μL of human target blood). Thus, from the addition of 0.72 μL infected Saimiri blood with 0.3% initial parasitaemia, there were estimated 10,800 infected RBCs per well. Given that there are 7 × 10^6^ RBCs/μL in Saimiri blood [85], and 5 × 10^6^ RBCs/μL in human blood, there would be a total of approximately 37 × 10^6^ RBCs in 7.2 μL, of which 10,800 were infected. Thus, the starting parasitaemia for the invasion experiment was estimated to be 0.03%.

The plate was maintained in a 10% CO_2_ incubator as above, and the medium was changed daily. Slides were made at 0, 72, and 144 h by resuspending each experimental well, and placing 1 μL of the resuspension on a glass slide. The slides were fixed in methanol and stained in Giemsa for microscopy as described above.

### Flow cytometry

At the 72 and 144 h time points, 25 μL of the resuspended in vitro *P. vivax* culture was incubated at 37°C for 30 min in 380 μL of flow cytometry stain. No extra Saimiri or human RBCs were added between 72 and 144 h. The flow cytometry staining solution [82, 86] was comprised of 5 μM Hoechst 33,342 (DNA stain, Invitrogen), 5 μM Pyronin Y (RNA stain, Sigma-Aldrich), and a FITC-conjugated Glycophorin A (CD235a) mouse anti-human monoclonal antibody (clone: CLB-ery-1 (AME-1), to differentiate human and Saimiri RBCs, Invitrogen), which was diluted 1:50 in 1 × PBS. After incubation, the stained in vitro culture was maintained on ice until flow imaging was complete with exposure to the UV 440, Yellow–Green 582, and Blue 525 lasers and filters on the Becton–Dickinson LSRII flow cytometer in the Cytometry & Imaging Microscopy Core Facility of the Case Comprehensive Cancer Center (CWRU). A total of 600,000 cells (events) per well were counted. The data was further analysed and gated using FCS Express 6 (De Novo Software).

### Cryopreservation and re-cultivation of cultured *Plasmodium vivax* patient isolate

From the second culture of isolate AMP2014.01 on day 69 post-culture, with parasitaemia at 0.35%, a 75 μL aliquot was mixed with an equal volume of Glycerolyte 57 solution and transferred into a 2-mL tube for cryopreservation. On day 5 post-cryopreservation, the cryopreserved aliquot was thawed and returned to culture using the same methods described above for the original patient isolate.

## Results

### Diagnosis of *Plasmodium* species infection in patient samples

All study patients exhibited symptoms of clinical malaria when they presented themselves to the Ampasimpotsy Health Centres from June 2014 to January 2017. Patient Ext30703 (AMP2014.01), a 15-year old male, presented with a fever of 38.0 °C, tested positive by malaria RDT and was slide-positive for *P. vivax* with a parasitaemia of 6320 parasites/µL (0.13%). Patient 1040902 (AMP2014.02), a 43-year old female, presented with a fever of 38.2 °C, tested positive by malaria RDT and was *P. vivax* slide-positive (1200 parasites/µL, 0.024%). Patient Ext2838 (AMP2016.01), a 3-year old male presented with a temperature of 36.7 °C, was malaria RDT positive and slide-positive for *P. vivax* (5509 parasites/µL, 0.11%). Patient Ext3276 (AMP2016.02), a 12-year old male, presented with a fever of 39.0 °C, was positive by malaria RDT and was slide-positive for *P. vivax* with a parasitaemia of 13,359 parasites/µL (0.27%). Molecular diagnosis using LDR-FMA [83] determined that all the above mentioned patient blood samples were positive only for *P. vivax* infection.

### In vitro culture initiation and growth dynamics of *Plasmodium vivax* patient isolates

Among the four patient isolates studied to-date, the median time interval between sample collection and in vitro culture was 454 days (range 166–961 days) (Table 1). The first in vitro culture for AMP2014.01 was initiated 666 days after collection; a second culture was initiated 961 days post-collection. In vitro culture for AMP2014.02, AMP2016.01 and AMP2016.02 was initiated 454, 298 and 166 days after collection, respectively.

**Table 1:** In vitro culture and growth characteristics of Malagasy *P. vivax* isolates

Isolate	Ampasimpotsy Health Center	Parasitaemia (parasitized RBCs/μL)	Culture initiation (days post- collection)	Culture duration (days)
2014.01 (first)	AMP Central	6320	666	233
2014.01 (second)			961	165
2014.02	AMP North	1200	454	155
2016.01	AMP South	5509	298	36
2016.02	AMP South	13,359	166	159

**Fig. 1:**
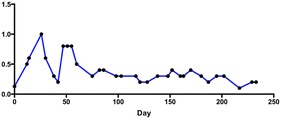
In vitro propagation of *Plasmodium vivax* patient isolate AMP2014.01. The fluctuation in growth of the parasites cultured in complete AIM V medium. Standard blood smear microscopy was performed to monitor parasite growth. Slides were made at intervals ranging from 2 to 15 days. Slides were made either after the medium was changed and the culture was resuspended, or right after the centrifugation to determine lysis and resuspending the pellet into fresh medium, but before adding Saimiri RBCs to readjust the haematocrit to 4%. With the patient parasitaemia of 0.13% on day 0, the culture parasitaemia fluctuated considerably during the first 60 days, and then maintained for the remaining culture period. DNA was extracted from cultured AMP2014.01 on days 86 and 202. PCR amplification and direct sequencing of *PvDBP* and *PvAMA*-*1* segments was performed at these time points to enable comparison between these sequences and those amplified from the infected patient blood sample prior to in vitro propagation

**Fig. 2:**
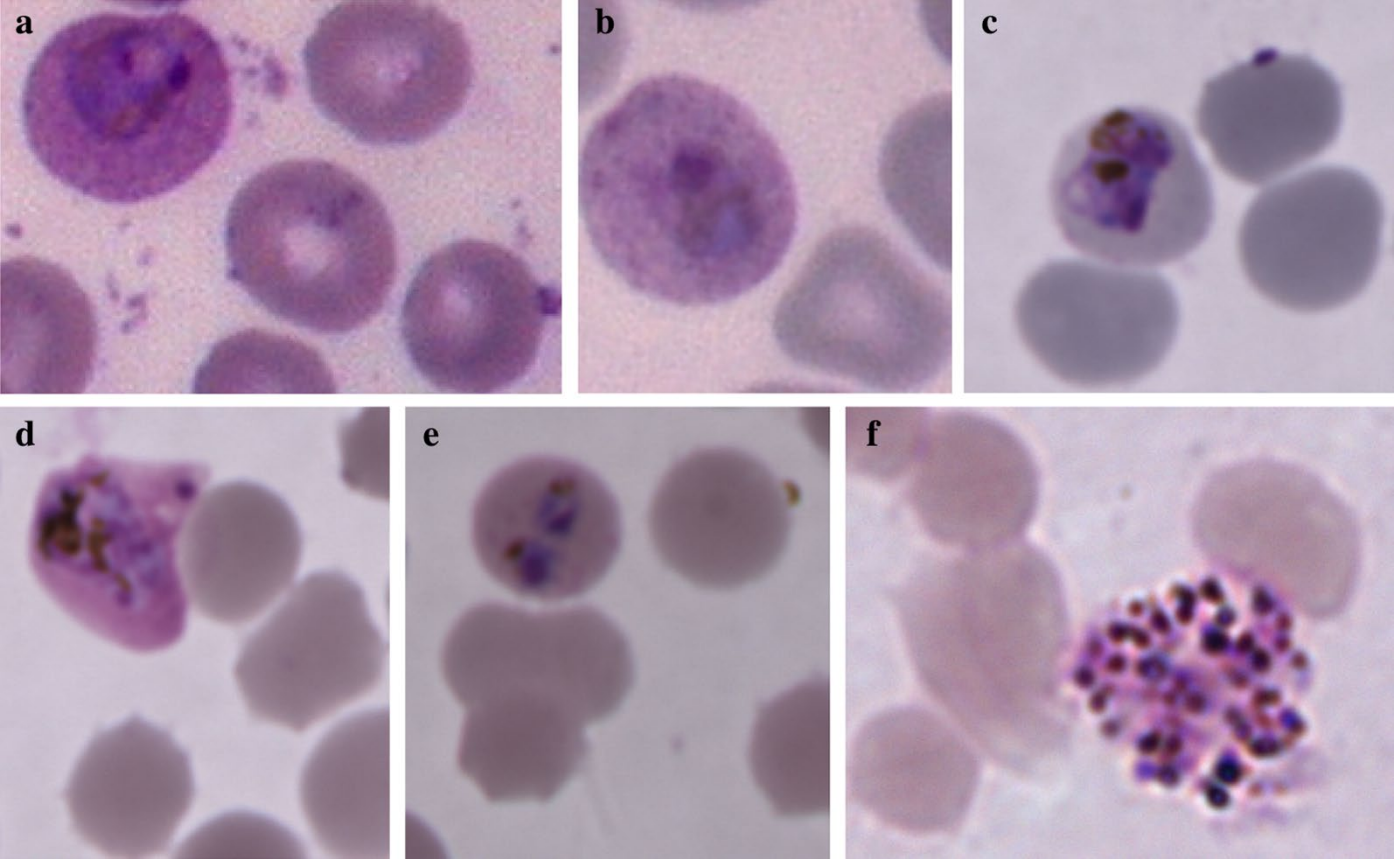
*Plasmodium vivax* AMP2014.01 blood smear images. **a**, **b** Trophozoite stages observed in the patient blood smear prepared at the time of sample collection. **c**–**f** show various infected Saimiri RBCs during 233 days of continuous in vitro culture; **c** young trophozoite (day 14); **d** maturing trophozoite (day 26); **e** doubly infected RBC (day 47), **f** Schizont (day 180)

Growth dynamics of isolate AMP2014.01, as monitored by Giemsa-stained thin smears, is presented in Fig. 1. Parasite density fluctuated between 0.2 and 1.0% (median 0.6%) during the first 60 days, and then maintained at 0.1–0.4% (median 0.3%) for the remaining in vitro culture period. At the beginning, the isolate was cultured in one 5-mL well for 71 days. Saimiri RBCs were added every 96–120 h to maintain 4% haematocrit. On day 72, 65% lysis (pellet volume 70 µL), instead of usual 50%, was observed. At that time, the culture was split into two 1-mL wells (24-well tissue culture plate) and the growth was further monitored. Isolate AMP2014.01 was in culture for 233 days, and was ended because of bacterial contamination. A second culture of this parasite was in continuous culture for 165 days (median parasitaemia 0.3%) and was terminated. From this second culture, human RBC invasion experiment was performed on day 25 post-culture, and cryopreservation and re-cultivation was attempted on day 69 post-culture (results below). Giemsa-stained trophozoite stages from the AMP2014.01 patient blood sample are shown in Fig. 2a, b. Additional parasite forms observed across the duration of the in vitro culture are shown in Fig. 2c–f. One may expect differences in the appearance of these parasite forms compared to what is possible from ex vivo samples (whether from humans, or nonhuman primates). Gametocytes were observed occasionally, but were not evaluated (counted) separately.

The median time in culture for the four isolates studied here has been 159 days (Table 1). AMP2014.02 and AMP2016.01 were in culture for 155 days (median parasitaemia 0.55%) and 36 days (median parasitaemia 0.2%), respectively. AMP2014.02 was terminated, whereas AMP2016.01 was ended because of bacterial contamination. AMP2016.02 was in continuous culture for 159 days (median parasitaemia 0.26%) and was terminated. Thus, it is evident that these parasites survived all stages of collection, cryopreservation, shipping, and thawing.

### Molecular characterization of cultured *Plasmodium vivax* patient isolates

On days 86 and 202, 40 μL of packed cells from one well of isolate AMP2014.01 were used to extract genomic DNA, which was subjected to molecular diagnosis using LDR-FMA and sequence analysis of the *PvDBP* and *PvAMA*-*1* genes. On these days, the estimated parasitaemias were 0.4 and 0.3%, respectively (Fig. 1). The LDR-FMA *Plasmodium* species diagnostic assay results confirmed the presence of only *P. vivax* in the culture.

DNA sequence analysis of *PvDBP* (518 bp) and *PvAMA*-*1* (395 bp) gene regions were performed to assess potential strain complexity of isolate AMP2014.01. These analyses are shown in comparison to the homologous sequences from the Sal-1, Chesson, and Palo Alto strains of *P. vivax* analysed as controls. Amino acid sequence comparisons are presented in Additional file 2: Figures S1 and S2, respectively. These results show that the *PvDBP* and *PvAMA*-*1* haplotype sequences from the cultures on days 86 and 202 (2014.01b and 2014.01c) were identical to those obtained from the patient blood sample (2014.01a). In addition, these results show that isolate AMP2014.01, collected from the Madagascar field site, carried *PvDBP* and *PvAMA*-*1* haplotypes that varied in numerous positions compared to the Sal-1, Chesson, and Palo Alto strains. Finally, each of the other three Malagasy *P. vivax* patient samples, collected from the same field site and subjected to in vitro culture, was characterized by unique *PvDBP* and *PvAMA*-*1* haplotypes. These observations technically exclude the possibility that the molecular diagnostic results might have been generated from the genomic DNA of control strains.

### Saimiri-adapted *Plasmodium vivax* invasion of human RBCs

**Fig. 3:**
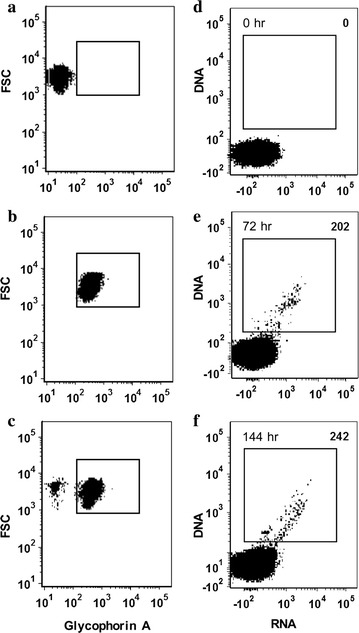
Identification and propagation of *Plasmodium vivax* AMP2014.01-infected human RBCs by flow cytometry. **a**–**c** The human glycophorin A (GPA)-specific monoclonal antibody (CD235a) was used to differentiate human RBCs from Saimiri RBCs separately and in mixed cultures. For experiments in **a**, **b** host RBCs were not exposed to AMP2014.01; infected Saimiri RBCs were introduced in the experiment shown in **c**. Assessment of FSC (Y-axis) in all panels further defines gating parameters of GPA− and GPA+ parasitized RBCs. **d**–**f** AMP2014.01-infected Saimiri RBCs were mixed with fresh human RBCs and their GPA+/DNA+ cells were identified (top right corner) at time 0, 72, and 144 h. Results show only those human GPA+ RBCs appearing within gating defined in **a**–**c**. All controls and experiments were run in triplicate

Following the adaptation of AMP2014.01 (second culture) to propagation in Saimiri RBCs for 25 days, it was investigated whether this isolate had retained the ability to invade human RBCs. Flow cytometry to assess the invasion of human RBCs was performed on cultures harvested at 72 and 144 h. The glycophorin A (GPA, CD235a) monoclonal antibody that stains human and not Saimiri RBCs was used to distinguish target from donor cells, respectively. Results in Fig. 3 compare binding of the GPA monoclonal antibody to Saimiri vs. human RBCs without (controls panels a and b, respectively), and with (panel c) parasites. These results illustrate the specificity of the GPA monoclonal antibody for human RBCs, and established the gating parameters to evaluate AMP2014.01 invasion of human RBCs (panels d–f).

Evaluation of the *P. vivax* propagation in human RBCs included addition of Hoechst 33342 (DNA stain) and Pyronin Y (RNA stain). Because RBCs do not contain a nucleus, uninfected RBCs would not stain with Hoechst 33,342. While human reticulocytes would retain residual RNA and stain positive for Pyronin Y, they would not stain positive for DNA with Hoechst 33342. Results (Fig. 3d–f) show DNA and RNA staining of GPA-positive RBCs, comparing time 0 h uninfected controls to 72 h and 144 h *P. vivax* infection time points. Given the 48-h in vivo replication cycle of *P. vivax*, the experimental time periods would represent 1.5–3 replication cycles. Three replicate cultures showed that the mean infected cell counts at 0 h was 0, at 72 h was 166 (range 112–202), and at 144 h was 211.3 (range 166–242). It was noted that there was a slight increase in the number of *P. vivax*-infected DNA+/RNA+ cells between the 72 and 144 h cultures (e.g., 202 to 242). Given that a total of 600,000 RBCs were counted by the flow cytometry, of which 540,000 were estimated to be human RBCs (parasite:target ratio 1:9), these 202 (72 h) and 242 (144 h) GPA-positive infected RBCs translate to 0.037 and 0.045% parasitaemias, respectively. Additionally, there appeared to be an increase in the RNA signal among infected cells in the 144 h culture, indicating cellular activity, which is consistent with previous studies of *P. falciparum* [82]. These results are consistent with a conclusion that, while propagated in Saimiri RBCs for a period of 25 days, isolate AMP2014.01 retained the capacity for invading and replicating in human RBCs.

Microscopic evaluation of the Giemsa-stained smears, made at 72 and 144 h from the wells showing 202 and 242 GPA-positive infected RBCs, showed that the corresponding parasitaemias were 0.1 and 0.2%, respectively. Infected human RBCs were not distinguished from infected Saimiri RBCs on these slides. Nevertheless, this evaluation confirms that new parasite invasion events occurred, as the starting parasitaemia for the invasion experiment was estimated to be 0.03% (see “Methods”).

### Cryopreservation and re-cultivation of cultured *Plasmodium vivax* patient isolate

The cryopreserved aliquot of cultured isolate AMP2014.01 was in continuous culture for 88 days and was terminated. The aliquot was cryopreserved at a parasitaemia of 0.35%. During the culture period, the parasitaemia ranged 0.1–0.4% (median 0.3%). These results suggest that this could be a successful cryopreservation and re-cultivation method for in vitro-generated parasites. These results appear to pave the way to stabilizing patient isolates and providing significant amounts of precious resources for further studies.

## Discussion

This study reports the progress made toward long-term in vitro culture of *P. vivax* isolates originating from infected patients in Ampasimpotsy, located in the Tsiroanomandidy Health District of Madagascar. This region has previously been shown to experience consistent transmission of *P. vivax* [63, 72, 87], including among Duffy-negative people [63]. Important first steps for understanding the genetic characteristics and biology of *P. vivax* parasites from this region require developing reliable methods for collecting, storing, transporting field-collected blood samples, and culturing parasites from those samples. All of the long-term in vitro culture work performed here has used whole blood from squirrel monkeys (*S. boliviensis*), without reticulocyte enrichment. These methods have reproducibly yielded long-term cultures of all four *P. vivax* isolates attempted so far (median time 159 days, range 36–233 days); none of the isolates failed to propagate using this culture system. This reproducibility was observed regardless of the anticoagulant, K^+^-EDTA or Na^+^-heparin, used in vacutainers to collect and store patient blood samples before cryopreservation. Furthermore, one of these isolates, AMP2014.01, demonstrated reproducible capacity for culture in Saimiri RBCs (two different long-term cultures have been initiated from separate cryopreserved blood sample vials), retained capacity for infecting human RBCs, and could be cryopreserved and re-cultivated.

### Cultivation of *Plasmodium vivax* patient isolates

The four *P. vivax* isolates cultured in this study were collected from patients with parasitaemias as low as 0.024% to as high as 0.27%. In addition, the time interval between sample collection and in vitro culture varied from 166 to 961 days. Despite this variation, a final pellet of intact RBCs between 30 to 80 μL was regularly observed when these patient samples were thawed and prepared for in vitro culture. The starting culture parasitaemias on day 0 from these thawed samples are likely to have been lower than the corresponding patient parasitaemias due to lysis during the thawing process. Thus, these methods proved to be efficient and reproducible in initiating cultures from small amounts of parasite material, and the success of culturing did not appear to be limited by low patient parasitaemia.

Along this line that these cultivation methods were efficient and reproducible in initiating cultures from low parasite material, cryopreservation and re-cultivation of the cultured parasites was achieved successfully. Seventy-five microliter of isolate AMP2014.01 (second culture) at a parasitaemia of 0.35% were cryopreserved. When this aliquot was thawed, a 20 μL pellet of intact RBCs was recovered and a culture in one 1-mL well was initiated. From this starting material, a parasitaemia of 0.4% was observed on day 33; the culture was split into two 1-mL wells on day 35, and was terminated on day 88.

An overall assessment of the growth dynamics of the patient isolates AMP2014.01 (first culture, 233 days; second culture, 165 days), AMP2014.02 (155 days), and AMP2016.02 (159 days) shows that the parasitaemia generally ranged 0.1–0.4%; for isolate AMP2014.02, it reached 1% twice during the culture period. A unique growth pattern was observed for isolate AMP2014.01 (first culture), where the parasitaemia fluctuated considerably during the first 60 days, and then maintained for the remaining culture period. This observation in particular is similar to those made by Roobsoong et al. [50] regarding patient isolates cultured in modified McCoy’s 5A medium with reticulocytes purified from adult peripheral blood added daily. In their culture system, parasite density was dramatically dropped during the first week of culture and then maintained at very low density for the whole culture period. Most of their 30 isolates shared a similar growth pattern with fluctuations of parasitaemia (Fig. 7b [50]).

As lysis (~ 50%) was observed in the culture every 96–120 h, Saimiri RBCs were added to maintain 4% haematocrit. The lysis did not appear to reflect expansion of the culture. Extensive breakage of Saimiri erythrocytes in the culture was reported previously by Lanners [45], which may reflect the extensive host-cell modifications that the parasite induces in the infected erythrocytes [88].

It is important to note that microscopic evaluation of the cultures was performed by counting a total of 1000 RBCs (10–20 100× fields, 50–100 RBCs/field) from 2 μL of resuspended cultures. Also, the intervals when the slides were made to evaluate parasitaemia were not regular, and ranged from 2 to 15 days. Average daily rate of change in parasitaemia of isolate AMP2014.01 (first culture) over 233 days culture period, divided into early (days 0–60, 10 observations) and later (days 75–233, 20 observations) periods, was estimated. Linear regression analysis showed that, with a patient parasitaemia of 0.13% at the initiation of the culture (day 0), the parasitaemia significantly increased by 0.064% (95% confidence interval 0.032–0.095, P = 0.001) per day during the early period. However, with parasitaemia reaching 0.3% on day 75, the parasitaemia did not significantly change during the later period. The growth dynamics of isolates AMP2014.01 (second culture) and AMP2016.02 were highly similar to that of isolate AMP2014.01 (first culture) during the later period. It is possible that counting more RBCs per slide and/or making slides to evaluate parasitaemia at a regular interval, every 48 h cycle of invasion and multiplication, may have provided a more accurate assessment of parasite growth dynamics and multiplication rate.

Finally, molecular characterization of the *PvDBP* and *PvAMA*-*1* segments was performed, which were amplified from all four patient isolates and AMP2014.01 (first culture) aliquots taken on days 86 and 202. This analysis provides some assessment of the genetic diversity in these samples, shows differences from the commonly used parasite strains, and proves that the AMP2014.01 patient isolate and culture aliquots carry the same haplotypes. Efforts to generate enough cultured packed cell volume required to carry out further genome-wide analysis are underway.

### Cross-species propagation

Given that long-term in vitro culture of *P. vivax* has been possible in RBCs from *S. boliviensis,* a question was whether this cross-species exposure eliminated the ability of *P. vivax* to invade human RBCs. The experimental results summarized in Fig. 3d–f, indicate that isolate AMP2014.01 retains the ability to invade human RBCs in the context of short-term invasion assays. Results that *P. vivax* strains exposed to Saimiri and Aotus RBCs do not lose their abilities to infect human RBCs are not unexpected given previously published results. Since the late 1980s, multiple monkey-adapted *P. vivax* strains (including Belem [41], Chesson [44], AMRU-1 [89] and Sal-1 [56]) have successfully been used under a number of different study designs to infect human RBCs.

While it is encouraging that Saimiri-adapted parasites were still able to invade human RBCs after 25 days in culture, it is acknowledged that this is a short time period. It remains to perform such studies using parasites which have been in culture for a longer period, and to investigate the variability of successful human RBC invasion among different patient isolates.

### Comparisons with previous approaches

Other groups have performed numerous well-designed investigations to improve in vitro culture of *P. vivax*. Given the robust productivity experienced with in vitro cultivation of the Malagasy *P. vivax* isolates in Saimiri RBCs, this system was compared to previous studies (Additional file 3: Table S2). Most of the previous studies have focused their efforts on long-term and short-term in vitro culture of *P. vivax* in human RBCs, with the notable exception of Mons et al. [90], who used Aotus RBCs for short-term growth of the parasite.

One previous study, by Lanners, most similar to the work performed here, attempted prolonged in vitro culture of the *P. vivax* Chesson strain in Saimiri RBCs and in a 1:1 mixture of Saimiri and human RBCs [45]. Further comparison with Lanners provides a contrast in methods and outcomes compared to the present study. In the discussion of his study, which concluded by sustaining low levels of *P. vivax* in vitro propagation for 16–22 days, Lanners commented that attempts to culture the parasite in Saimiri RBCs and Saimiri serum were abandoned owing to the fragility of the monkey RBCs. His continued ability to maintain his cultures was the result of providing human reticulocytes and 15% human AB serum. Lanners’ work and many other earlier studies used RPMI 1640 as the base of their culture medium. Maintaining *P. vivax* laboratory as well as Malagasy patient isolate cultures using RPMI 1640 has been very difficult; our those efforts were marked by inconsistent propagation and early termination of cultures (Grimberg and Zimmerman, unpublished observations). Methods from other recent studies have also shown evidence of moving away from this medium (Additional file 3: Table S2). In addition to the use of AIM V medium, the cultures in the present study were supplemented with a lower concentration (10%) of heat-inactivated human AB serum than most other studies (range from 10 to 50%; Additional file 3: Table S2). Finally, the gas mixture used was 10% CO_2_. AIM V medium formulation is proprietary and has not been disclosed by the manufacturer. However, as mentioned previously, the base medium for AIM V is DMEM (manufacturer’s notes). Since the content of NAHCO_3_ in the culture medium determines the CO_2_ concentration for culturing, 10% CO_2_ was used in the present study (NAHCO_3_ content in DMEM, 3.7 g/L). In comparison, the NAHCO_3_ content of RPMI 1640 is 2.0 g/L, therefore 5% CO_2_ is used.

The preference *P. vivax* displays for reticulocytes has been demonstrated by both in vivo [52, 54, 55] and in vitro observations [41, 50, 51, 55, 56]. Acknowledging the challenges of *P. vivax* in vitro culture [40–49], together with previous experience [91], reticulocyte enrichment was investigated. Those efforts included strategies that preferentially lyse older RBCs [92], as well as strategies that separate reticulocytes from older RBCs by centrifugation through Percoll or selection by CD71 positivity [47, 51]. Overall, these methods have produced variable results. The in vitro cultivation method evaluated here has not included reticulocyte enrichment. One reason for not including reticulocyte enrichment was the small volume (2–3 mL [maximum]) of Saimiri blood requested every 2 weeks. Studies that have performed reticulocyte enrichment to culture *P. vivax* have used larger volumes of blood [44, 50, 56]. Interestingly, the lack of reticulocyte enrichment has not appeared to hamper adaptation of the Malagasy *P. vivax* isolates to in vitro propagation in Saimiri blood. Saimiri blood contains about 2% reticulocytes [85]. However, it is believed that reticulocyte counts in Saimiri blood, stored at 4 °C, are not available. In the present study, in the leukocyte-depleted Saimiri blood preparations, stored at 4 °C, reticulocyte counts were stable at about 2% over a 2-week period, which is when the fresh blood was obtained. This is in accordance with the results of a study on leukocyte-depleted human blood, stored for 6 weeks under standard blood bank conditions (2–6 °C) [93]. In this study, reticulocyte counts increased at day 21 and remained constant over time. Finally, recent reports have demonstrated a preference of the parasite for young reticulocytes, including that *P. vivax* Sal-1 rings ex vivo were found in young reticulocytes [56]. However, in this same study, the second generation (20 h in vitro) rings and trophozoites were found in older reticulocytes and mature RBCs [56]. Whether the storage of Saimiri blood, containing reticulocytes, for 2–3 weeks affects the invasion and maturation of the *P. vivax* isolates remains to be explored. For now, avoiding reticulocyte enrichment has saved considerable time and resources, and appears to simplify maintenance of *P. vivax* cultures in the present system.

### Limitations and future directions

Despite the experience of maintaining in vitro culture of multiple *P. vivax* isolates from Madagascar in Saimiri RBCs, the focus here on isolate AMP2014.01 encounters some limitations. The authors recognize that they have not performed the same manipulations demonstrated for AMP2014.01 with all of the isolates introduced in this study. Similarly, studying the receptivity of different *P. vivax* isolates to cryopreservation and onward rounds of in vitro culture will need to be performed. Such studies would further substantiate if the approaches described here are able to successfully contribute to the development of stable parasite strains that are characterized by distinct biological phenotypes from Madagascar and other endemic areas.

Beyond demonstrating the basic capacity of *P. vivax* in vitro culture in Saimiri RBCs, it will be important to investigate further if this system can be used to characterize the specific factors involved in *P. vivax* RBC invasion, as has been performed by laboratory-adapted strains of *P. falciparum* [33, 94, 95]. Therefore, a number of in vitro studies are readily anticipated. These include tests to determine if treatments to manipulate the RBC surface (e.g., trypsin, chymotrypsin, neuraminidase) and antibody reagents interacting with the Duffy blood group and Duffy binding protein affect parasite RBC invasion in predictable ways. Beyond this basic assessment of *P. vivax* in an in vitro setting, it will be exciting to determine if the methods described here will facilitate a wider range of investigations into the lifecycle of this parasite to include attaining gametocytes, exposure of mosquito hosts to different *P. vivax* strains, evaluation of liver-stage infection and development of hypnozoites. These studies, as well as analyses of gene expression and genetic manipulation of this parasite would all be facilitated if it is possible to develop stable, laboratory-adapted strains of *P. vivax*. Finally, investigation of the possible differences in expression/biology of the parasite grown in Saimiri *vs.* human RBCs would also be facilitated.

## Conclusions

Long-term culture of *P. vivax* is possible in RBCs of *Saimiri boliviensis*. Past studies have shown that *S. boliviensis* serves as a receptive in vivo host [57, 66–70]. The studies here introduce an alternative to propagation of *P. vivax* in live animals, which is becoming more restrictive. In vitro culture of *P. vivax* in Saimiri RBCs provides new opportunities for investigating the molecular and cellular biology of this important malaria parasite.

## Additional files

**Additional file 1: Table S1.** PvDBP and PvAMA-1 primer sequences.

**Additional file 2: Figure S1.** *PvDBP* sequence comparison. **Figure S2.** *PvAMA-1* sequence comparison

**Additional file 3: Table S2.** Comparing methods for long-term and short-term in vitro culture of *Plasmodium vivax* (Adapted from Noulin, 2013).
