# Supplementary material for: Long-term in vitro culture of Plasmodium vivax isolates from Madagascar maintained in Saimiri boliviensis blood
Source: Malar J. 2017 Nov 3;16:442. doi: 10.1186/s12936-017-2090-7 (PMC5670718; doi:10.1186/s12936-017-2090-7)
Supplement: Supplementary file 2 — Additional file 2: Figure S1. PvDBP sequence comparison. Figure S2. PvAMA-1 sequence comparison [file 12936_2017_2090_MOESM2_ESM.docx]

Supplemental Figure 1: *PvDBP* sequence comparison

Sal-1 NFHRDITFRKLYLKRKLIYDAAVEGDLLLKLNNYRYNKDFCKDIRWSLGD

Chesson --------------------------------------------------

Palo Alto ---S------------------------F---------------------

2014.01a --------------------------------------------------

2014.01b --------------------------------------------------

2014.01c --------------------------------------------------

2014.02a --------------------------------------------------

2016.01a --------------------------------------------------

2016.02a --------------------------------------------------

Sal-1 FGDIIMGTDMEGIGYSKVVENNLRSIFGTDEKAQQRRKQWWNESKAQIWT

Chesson -----------------------------G-----H--------------

Palo Alto --------------------D--------GKN---H--------------

2014.01a ----------------E------------G-Q------------------

2014.01b ----------------E------------G-Q------------------

2014.01c ----------------E------------G-Q------------------

2014.02a -----------------------------G-----H--------------

2016.01a -----------------------------G-----H--------------

2016.02a ----------------E------------G-Q------------------

Sal-1 AMMYSVKKRLKGNFIWICKLNVAVNIEPQIYRWIREWGRDYVSELPTEVQ

Chesson --------------------------------------------------

Palo Alto ------------K------I------------R-----------------

2014.01a ------------K------I------------R-----------------

2014.01b ------------K------I------------R-----------------

2014.01c ------------K------I------------R-----------------

2014.02a --------------------------------------------------

2016.01a --------------------------------------------------

2016.02a --------------------------------R-----------------

Sal-1 KLKEKCDGKINYTDKKVCKVPP

Chesson ----------------------

Palo Alto ----------------------

2014.01a ----------------------

2014.01b ----------------------

2014.01c ----------------------

2014.02a ----------------------

2016.01a ----------------------

2016.02a ----------------------

**Legend – *PvDBP* region 2 sequence comparisons.** Alignments compare *PvDBP* region 2 amino acid sequences for monkey-adapted *P. vivax* strains (Sal-1, Chesson, and Palo Alto), Malagasy field isolates, and the resulting *in vitro* culture strains. Complete records including *PvDBP* DNA sequence are accessible through GenBank accession numbers KX576687, KY794603, KY794602, and KY861926 for AMP2014.01, AMP2014.02, AMP2016.01, and AMP2016.02, respectively. Sequences AMP2014.01a, AMP2014.01b, and AMP2014.01c are from the patient blood sample, Day 86, and Day 202 *in vitro* cultures, respectively. AMP2014.02a, AMP2016.01a, and AMP2016.02a are from the patient blood samples.

Supplemental Figure 2: *PvAMA-1* sequence comparison

Sal-1 RPVATGDQKLKDGGFAFPNANDHISPMTLANLKERYKDNVEMMKLNDIAL

Chesson --------R---------K-D-----------------------------

Palo Alto --------R--------------------E---A----------------

2014.01a --------R--------------------E---A----------------

2014.01b --------R--------------------E---A----------------

2014.01c --------R--------------------E---A----------------

2014.02a --------------------------------------------------

2016.01a --------R--------------------E---A----------------

2016.02a --------------------------------------------------

Sal-1 CRTHAASFVMAGDQNSSYRHPAVYDEKEKTCHMLYLSAQENMGPRYCSPD

Chesson --------------------------------------------------

Palo Alto --------------------------------------------------

2014.01a ---------------------------K--------------------S-

2014.01b ---------------------------K--------------------S-

2014.01c ---------------------------K--------------------S-

2014.02a --------------------------------------------------

2016.01a -------------------------------Y------------------

2016.02a --------------------------------------------------

Sal-1 AQNRDAVFCFKPDKNESFENLVYLSKNVRND

Chesson -------------------------------

Palo Alto -------------------------------

2014.01a -------------------------------

2014.01b -------------------------------

2014.01c -------------------------------

2014.02a -------------------------------

2016.01a -------------------------------

2016.02a ------------HE-----------------

**Legend – *PvAMA-1* region 1 sequence comparisons.** Alignments compare *PvAMA-1* region 1 amino acid sequences for monkey-adapted *P. vivax* strains (Sal-1, Chesson, and Palo Alto), Malagasy field isolates, and the resulting *in vitro* culture strains. Complete records including *PvAMA-1* DNA sequence are accessible through GenBank accession numbers KX553965, KY794601, KY794600, and KY794587 for AMP2014.01, AMP2014.02, AMP2016.01, and AMP2016.02, respectively. Sequences AMP2014.01a, AMP2014.01b, and AMP2014.01c are from the patient blood sample, Day 86, and Day 202 *in vitro* cultures, respectively. AMP2014.02a, AMP2016.01a, and AMP2016.02a are from the patient blood samples.
